# *TGFBR1* Intralocus Epistatic Interaction as a Risk Factor for Colorectal Cancer

**DOI:** 10.1371/journal.pone.0030812

**Published:** 2012-01-23

**Authors:** Ana Martinez-Canto, Adela Castillejo, Trinidad Mata-Balaguer, Maria-Isabel Castillejo, Eva Hernandez-Illan, Esperanza Irles, Victor Manuel Barbera, Cecilia Egoavil, Carla Guarinos, Cristina Alenda, Enrique Ochoa, Rafael Lazaro, Silvia Fajardo, Javier Lacueva, Rafael Calpena, Jose Luis Soto

**Affiliations:** 1 Molecular Genetics Laboratory, Elche University Hospital, Elche, Spain; 2 Department of Pathology, Alicante University Hospital, Alicante, Spain; 3 Molecular Biopathology, Castellon Provincial Hospital, Castellon, Spain; 4 Department of Pathology, La Plana Hospital, Villareal, Spain; 5 Department of Surgery, Elche University Hospital, Elche, Spain; Harvard School of Public Health, United States of America

## Abstract

In colorectal cancer (CRC), an inherited susceptibility risk affects about 35% of patients, whereas high-penetrance germline mutations account for <6% of cases. A considerable proportion of sporadic tumors could be explained by the coinheritance of multiple low-penetrance variants, some of which are common. We assessed the susceptibility to CRC conferred by genetic variants at the *TGFBR1* locus. We analyzed 14 polymorphisms and the allele-specific expression (ASE) of *TGFBR1* in 1025 individuals from the Spanish population. A case-control study was undertaken with 504 controls and 521 patients with sporadic CRC. Fourteen polymorphisms located at the *TGFBR1* locus were genotyped with the *iPLEX Gold* (*MassARRAY-Sequenom*) technology. Descriptive analyses of the polymorphisms and haplotypes and association studies were performed with the SNPator workpackage. No relevant associations were detected between individual polymorphisms or haplotypes and the risk of CRC. The TGFBR1*9A/6A polymorphism was used for the ASE analysis. Heterozygous individuals were analyzed for ASE by fragment analysis using cDNA from normal tissue. The relative level of allelic expression was extrapolated from a standard curve. The cutoff value was calculated with Youden's index. ASE was found in 25.4% of patients and 16.4% of controls. Considering both bimodal and continuous types of distribution, no significant differences between the ASE values of patients and controls were identified. Interestingly, a combined analysis of the polymorphisms and ASE for the association with CRC occurrence revealed that ASE-positive individuals carrying one of the most common haplotypes (H2: 20.7%) showed remarkable susceptibility to CRC (RR: 5.25; 95% CI: 2.547–5.250; p<0.001) with a synergy factor of 3.7. In our study, 54.1% of sporadic CRC cases were attributable to the coinheritance of the H2 haplotype and *TGFBR1* ASE. These results support the hypothesis that the allelic architecture of cancer genes, rather than individual polymorphisms, more accurately defines the CRC risk.

## Introduction

Colorectal cancer (CRC) affects more than one million people worldwide every year and is becoming the most prevalent type of cancer in developed countries [1]. The underlying causes of CRC are combinations of environmental and genetic factors in different proportions. A considerable percentage of sporadic tumors could be explained by the coinheritance of multiple low-penetrance variants, some of which are common. Inherited susceptibility underlies ∼35% of the variance in CRC risk, whereas high-penetrance germline mutations account for <6% of cases [2].

Common genetic variants at several loci involved in the transforming growth factor beta (TGF-beta) superfamily signaling pathway have been identified as low-penetrance variants that affect CRC development, when an unbiased approach is used, such as a genome-wide association (GWA) analysis [2].

TGF-beta is one of the most potent inhibitors of the proliferation of epithelial cells. Abnormalities in this signaling pathway are almost universal in cancer cells and are mediated through a variety of mechanisms [3]. The TGF-beta receptor type I (encoded by the *TGFBR1* gene) is a mediator of TGF-beta growth-inhibitory signals and has been targeted in several studies of cancer susceptibility and progression, with frequently discordant results [4]–[7].

Recently, a phenomenon called “allele-specific expression” (ASE) was described; ASE occurs in the germline at the *TGFBR1* gene in 10%–20% of CRC patients and generates an increased risk of CRC (odds ratio [OR]: 8.7; 95% confidence interval [CI]: 2.6–29.1), although the underlying genetic cause of this transcriptional variation remains unknown [8]. More recently, contrary results were reported, in that the ASE of *TGFBR1* was observed as a rare event and no increased susceptibility to CRC could be detected [9]–[14].

It is currently accepted that there is a direct association between a genetic susceptibility to cancer and the number of risk alleles carried by an individual [15]. Evidence for this assumption comes from several studies in which the authors analyzed a combination of a small number of susceptibility alleles at different loci [2]. The 2% of the population with the highest risk, who carried multiple low-risk alleles, had an increase in CRC of about fourfold compared with individuals with a median population risk [15].

In the present study, we aimed to map the genetic susceptibility interactions for CRC at the *TGFBR1* locus. Our results show that individuals carrying the combination of a specific haplotype and ASE have a substantially increased risk of CRC (relative risk [RR]: 5.25; 95% CI: 2.547–5.250; p<0.001), although neither of these factors had a significant effect on CRC susceptibility when analyzed individually.

## Methods

### Objectives

The working hypothesis we tested in this study was that the detailed intralocus allele architecture of *TGFBR1* more precisely predicts genetic susceptibility to CRC than do individual single-nucleotide polymorphisms (SNPs). We aimed to map the genetic susceptibility interactions at the *TGFBR1 locus* that affect CRC, defined by 14 polymorphisms and *TGFBR1* ASE, in a case-control study.

### Participants

#### Patients with sporadic CRC

Individuals with sporadic CRC (n = 521) who underwent surgery with curative intention were included in this study. Patients with familial adenomatous polyposis or Lynch syndrome were excluded. Patients suspected of having Lynch syndrome (tumors diagnosed earlier than 50 years of age, with microsatellite instability) were also excluded. The median age of the included patients at diagnosis was 67 years (range 23–93 years). The clinical and pathological characteristics of the CRC patients are given in detail in Table 1.

**Table 1:** Clinical and pathological characteristics of CRC patients.

Variables	Frequencies	
**Sex**		
Men	290	55.66%
Women	231	44.34%
**Age**		
Median	67	
Range	23–93	
**Location**		
Proximal	98	24.08%
Distal	309	75.92%
UK	114	
**Stage**		
I	8	2.74%
II	133	45.55%
III	146	50.00%
IV	5	1.71%
UK	229	

(UK: unknown).

Patients' biological samples and clinical and pathological information were obtained from the Biobanks at Elche University Hospital and Castellon Provincial Hospital (Spain).

#### Controls

Controls (n = 504) with no personal history of cancer and with diagnoses thought to be unrelated to the disease of interest (e.g., bone fractures, multiple trauma, blood glucose irregularities, vascular and heart diseases, complications associated with renal failure) were selected from the Elche University Hospital Biobank (Spain). Their median age was 72 years (range 23–98 years).

### Description of procedures and investigations undertaken

#### DNA/RNA extraction and cDNA synthesis

DNA and RNA were extracted from the peripheral blood leukocytes of the controls and from the normal-appearing colonic mucosa of the CRC patients. DNA/RNA extraction and cDNA synthesis were performed as described previously [16].

#### Polymorphism selection

The selection of SNPs was based on their association with the occurrence of *TGFBR1* ASE, as described by Valle et al. [8]. A total of 14 polymorphisms extending along 71 kb at the *TGFBR1 locus* were genotyped. Six SNPs were intragenic, five were located upstream from the gene, and three were located downstream from the gene (Figure S1). The SNPs rs7034716 and rs6478974 are described as tagSNPs in this region, according to the HapMap database (Data Rel27 Phase II+III).

#### SNP genotyping

MassARRAY Designer software (Sequenom) was used to design the PCR assay and iPLEX single-base extension primers for the multiplexed analysis. iPLEX Gold assay MassARRAY (Sequenom) is a primer extension process designed to detect sequence differences at the single-nucleotide level. Allele-specific differences in mass between extension products are detected by MALDI-TOF/MS.

#### Analysis of the *TGFBR1* 9A/6A polymorphism

We have previously reported the genotyping of the polymorphic repetitive trinucleotide 9A/6A in *TGFBR1* exon 1 (rs11466445) in patients with sporadic CRC and in controls [16].

#### ASE analysis

We used cDNA from all the heterozygous 9A/6A individuals to analyze the ASE of *TGFBR1*. To quantify the ASE, we used a nine-point standard curve constructed with dilutions of cDNAs from 9A and 6A homozygous individuals dilutions: 9∶1, 8∶2, 7∶3, 6∶4, 5∶5, 4∶6, 3∶7, 2∶8, and 1∶9). A standard curve (Pearson's correlation coefficient >0.98) was used to interpolate the relative ASE for each individual. Each cDNA sample was tested in triplicate. cDNA samples from three heterozygous individuals were also used as calibrators throughout all the quantitative ASE experiments to evaluate and correct for potential interexperimental variation. The cutoff value was calculated with a receiver operating curve analysis, to estimate the sensitivity and specificity of the various cutoff points, and to select the best value for Youden's index.

### Ethics

Written informed consent for inclusion in the respective Biobank was obtained from every participating individual. The study was approved by the ethics committees of the Elche and Castellon Hospitals.

### Statistical methods

Descriptive analyses of the polymorphisms and haplotypes were performed with the genetic statistical platform SNPator [17]. Before the individual polymorphisms were analyzed, Hardy-Weinberg equilibrium was confirmed for the control group. The SNPator platform uses the PHASE program for haplotype estimation. This program implements a Bayesian statistical method for reconstructing haplotypes from population genotype data.

Multivariate unconditional logistic regression models assuming dominant, recessive, additive, or codominant modes of inheritance were used to assess the associations between the polymorphisms or haplotypes and CRC.

We explored the potential effects of modification by sex, age (below vs over the median age: 67 years), tumor location (proximal vs distal), and tumor stage (I and II vs III and IV) in the corresponding stratified analysis.

A χ^2^ test was used to evaluate the differences in variant carrier frequencies between the patients group and the control group and to analyze any association between the SNPs and the clinical and pathological factors. Armitage's trend test was used to calculate p for trends in the additive model of inheritance. All p values were two-sided and p<0.05 was considered significant. The results are expressed as ORs and 95% CIs. Power was determined using online statistical software (http://www.stat.ubc.ca/~rollin/stats/ssize/caco.html). Bonferroni's method to correct for multiple tests was included in the analysis to ensure that the overall confidence coefficient was maintained. A nonparametric Mann-Whitney U test was used for the ASE analysis when the distribution was considered continuous. The RR, synergy factor, and population attributable risk percent (PAR%) were calculated in the combined analysis of haplotypes and ASE.

## Results

There was no statistically significant difference in the mean age or sex distribution of the patients and controls

### TGFBR1 polymorphisms and CRC

A total of 782 individuals (405 patients and 377 controls) were genotyped for the 13 SNPs. The amount and/or quality of the DNA of the remaining individuals were inadequate for analysis with the iPlex technology. Quality control for genotyping was assessed by real-time PCR with TaqMan probes for allelic discrimination in 0.7% of the genotypes identified by iPLEX technology. Genotyping of the polymorphic repetitive trinucleotide 9A/6A in *TGFBR1* exon 1 (rs11466445) was assessed in all patients and controls included in the study.

The allelic and genotype frequencies are shown in Tables S1 and S2, respectively. All the polymorphisms included in this study had a minor allele frequency (MAF) higher than 8% (Tables S1 and S2). The genotype distribution in the control population did not deviate significantly from that expected for a population in Hardy–Weinberg equilibrium (*P*>0.25). The allelic frequencies found were similar to those reported in the HapMap database (http://hapmap.org) and the NCBI dbSNP (http://www.ncbi.nlm.nih.gov/snp/).

Association studies of individual SNPs with CRC occurrence showed significant results for SNPs rs7034716, rs10739778, and rs334365, with ORs of 1.36–1.42 (Table 2). A stratified analysis showed a significant association between the minor allele and a CRC diagnosis at a younger age (<67 years) for the SNPs rs7033283, 7034462, 7034867, 12686783, 11466445, and rs928180 (Table 3). No other significant association was found when the analysis was stratified by sex, tumor location, or stage.

**Table 2:** Significant associations between individual *TGFBR1* polymorphisms and crude CRC occurrence.

Polymorphism	Genotype frequencies		OR	95% CI	*P*
**rs7034716**					
	CT	CC+TT			
CRC	148	255	1.420	(1.06–1.90)	0.0174
C	164	199			
**rs10739778**					
	AC	AA+CC			
CRC	170	235	1.360	(1.02–1.81)	0.033
C	184	187			
**rs334365**					
	AG	AA+GG			
CRC	161	235	1.404	(1.05–1.87)	0.0209
C	176	183			

(CRC: colorectal cancer; C: controls; OR: odds ratio; CI: confidence interval).

**Table 3:** Significant associations between individual *TGFBR1* polymorphisms and CRC stratified by age at diagnosis (<67 years).

Polymorphism	MAF	OR	95% CI	*P*
rs7033283	A	0.523	(0.29–0.95)	0.0325
rs7034462	T	0.526	(0.29–0.96)	0.0340
rs7034867	A	0.560	(0.32–0.99)	0.0432
rs12686783	T	0.576	(0.32–1.05)	0.0375
rs11466445	*6A	0.520	(0.29–0.92)	0.0288
rs928180	G	0.439	(0.22–0.87)	0.0157

(MAF: minor allele frequency; OR: odds ratio; CI: confidence interval).

A linkage disequilibrium (LD) study showed a generally high level of linkage between the polymorphisms analyzed. The linkage values for more than 80% of pairs of SNPs resulted in D′>0.8. The identified tagSNPs for this region included the SNPs rs7034462, rs7034867, and rs928180, all with MAF<10% (Table S3).

A haplotype analysis showed the presence of 17 and 27 different haplotypes in the controls and patients, respectively. Only six of those haplotypes (H1, H2, H3, H4, H5, and H13) had a frequency higher than 1% in the controls; these were selected for the association studies. Descriptions of the haplotypes and their frequencies are given in Tables S4 and S5, respectively.

Some significant associations between the haplotypes and CRC were found in the stratified analysis. For individuals aged over 67 years, the H2 and H5 haplotypes were associated with an increase and a reduction in CRC, respectively. Individuals aged below 67 years and carrying the H1 haplotype had a lower risk of CRC. Finally, male individuals carrying the H4 haplotype had a lower risk of CRC (Table 4).

**Table 4:** Significant associations between *TGFBR1 locus* haplotype and CRC stratified by age at diagnosis and sex.

	CRC	C	OR	95% CI	*P*
**Age**					
>67 years					
Haplotype H2	n = 183	n = 225			
Carriers	73	64			
Non carriers	110	161	1.669	(1.10–2.52)	0.015

(CRC: colorectal cancer; C: controls; OR: odds ratio; CI: confidence interval).

### *TGFBR1* ASE

**Figure 1:**
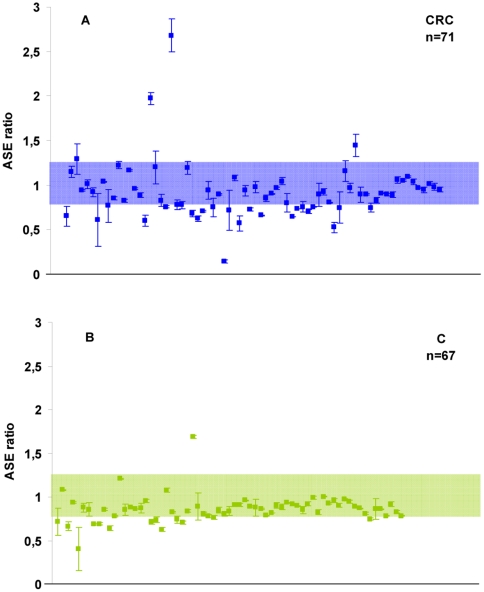
Relative expression of the *TGFBR1* rs11466445 allele calculated from a standard curve. The *TGFBR1* allele ratios in patients with sporadic CRC (panel A) and controls (panel B). The ASE-negative area is represented by the colored rectangle between the cutoff values (0.78 and 1.27). The mean and standard deviation are shown for each sample.

An ASE analysis was performed on the informative individuals for the rs11466445 polymorphism: 9A/6A heterozygous individuals (71 patients and 67 controls; n = 138). The relative *TGFBR1* expression calculated from a standard curve was plotted (Figure 1). The median ASE ratio was slightly higher for the CRC patients than for the controls (0.896±0.317 vs 0.862±0.155, respectively).

For the analysis in which ASE was considered to have a bimodal type of distribution, individuals were considered positive for the presence of ASE if they demonstrated an allelic expression ratio <0.78 or >1.27. These cutoff values corresponded to 9A/6A allele proportions of 44∶56 and 56∶44, respectively (sensitivity: 0.295; specificity: 0.836; Youden's index: 0.131; Table S6). In the positive patients, a consistent relative overexpression of the *6A allele was observed (*P* = 0.045).

Eighteen patients (25.4%) and 11 controls (16.4%) were positive for ASE, showing no significant association with cancer risk (OR: 1.697; 95% CI: 0.74–3.87; *P* = 0.213). In the stratified analysis of CRC, ASE was more frequently found in individuals with early-stage tumors (*P* = 0.016; Table S7).

No significant association was found when ASE was considered a continuous variable (*P* = 0.179).

### Combined analysis of TGFBR1 ASE and haplotypes

We found a highly significant association between the second most common haplotype H2 (patients: 24.07%; controls: 20.72%), ASE (patients: 25.4%; controls: 16.4%), and CRC occurrence. Individuals carrying the H2 haplotype with ASE showed a high risk of CRC (*P*<0.0001; Table 5). When H2 and ASE were considered independent factors, the frequencies observed in the patients differed significantly from the expected frequencies (*P* = 0.013), but did not do so in the controls (*P*>0.05; Table S8).

**Table 5:** Association between *TGFBR1* H2 haplotype and *TGFBR1* ASE.

Haplotype H2 carriers	CRC	C	OR: inf; 95% CI:(6.92-inf)
ASE positive	8	0	**RR: 5.250; 95% CI:(2.547–5.250)**
ASE negative	4	17	***P*** **<0.0001**

(CRC: colorectal cancer; C: controls; RR: relative risk; CI: confidence interval).

The RR values for ASE and the H2 haplotype were 1.24 (95% CI: 0.86–1.68; *P* = 0.213) and 1.123 (95% CI: 0.98–1.28; *P* = 0.095), respectively. As a consequence, the expected combined RR for ASE and H2 was 1.42, whereas the observed RR was 5.250 (95% CI: 2.55–5.25; *P*<0.0001). The resulting synergy factor was 3.7 and the population attributable risk was 54.1% (Table 6).

**Table 6:** Association between the *TGFBR1* H2 haplotype, *TGFBR1* ASE, and CRC.

	CRC	C	RR (95% CI)	*P*
**ASE**				
Carriers	18	11		
Non carriers	54	56	1.264 (0.86–1.68)	0.213
**H2**				
Carriers	173	139		
Non carriers	232	238	1.123 (0.98–1.28)	0.095
Carriers of H2+ASE	8	0		
Non carriers of H2+ASE	4	17	5.250 (2.55–5.25)	<0.0001
Expected RR	1.42			
Synergy factor	3.7			
PAR%	54.1%			

(CRC: colorectal cancer; C: controls; ASE: allele-specific expression; RR: relative risk; CI: confidence interval; PAR%: population attributable risk percent).

## Discussion

According to our results, the combined analysis of multiple genetic factors associated with cancer susceptibility, with insubstantial individual weights, might reveal a more precise intralocus allelic architecture than can individual analyses, and define specific subgroups of individuals with important levels of risk for CRC.

In the current work, we have presented evidence that individuals carrying the specific *TGFBR1* H2 haplotype and *TGFBR1* ASE have a high relative risk of CRC (RR = 5.250; 95% CI: 2.55–5.25), whereas the individual risk associated with each of these factors is negligible. A considerable synergistic relationship was identified in the combined analysis, lending support to the hypothesis that the allelic architecture of cancer genes might more accurately define the cancer risk. The calculated PAR% was 54.1%, indicating that more than half the sporadic CRC in our population was attributable to the combination of the H2 and ASE genetic factors at the *TGFBR1 locus*. Further independent studies with larger samples are required to confirm these results.

It is currently accepted that the effect of most common low-penetrance alleles is essentially independent and the possession of an increased number of risk alleles is associated with an increased cancer risk, consistent with a polygenic model of disease susceptibility [15].

Our results suggest that intralocus epistatic interactions between common variants of CRC susceptibility factors might exist. Direct experimental evidence of the existence of mechanisms for such intralocus epistatic interactions has been reported previously [18].

Modest gene expression changes can have significant biological consequences, as seen in *APC* gene, where 50% constitutional reductions in the expression of one allele can lead to the development of familial adenomatous polyposis [19].

ASE is a widespread phenomenon affecting the expression of 20% of human genes. The allelic differences are heritable in an autosomal manner and are not imprinted [20], [21]. Defining ASE will help us to appreciate the extent of functionally important regulatory variations and to focus on candidate haplotypes that are associated with variably expressed alleles, allowing the detailed molecular characterization of specific polymorphisms [20].

ASE represents the phenotypic effect of unknown genetic variations, and may involve one or more local or distant factors, which may act in *cis* or *trans*. Moreover, cumulative effects, epistasis, genotype-environment interactions, and pleiotropy may account for the complex genetic architecture underlying these transcriptional variations [22]. Plausible heterogeneity in regulatory factors, differences in selected cohorts of CRC patients, and differences in methodological approaches may be responsible for the apparently incongruent results published for *TGFBR1* ASE [8]–[14].

ASE has been measured by several authors by calculating the dosage ratio (cDNA/gDNA) [8], [10]–[12]. However, it is recognized that this methodology may entail occasional inherent problems with quantitative genotyping [9]. We decided that ASE should be assessed relative to the hypothetical 1∶1 ratio of the allelic dose rather than by comparing the cDNA and gDNA doses in a single sample. The relative quantification of the expression of each allele by extrapolation from a standard curve might offer more precise results [9], [23]. An additional possible confounding factor for ASE assessment could be the RNA source used. Lymphoblastoid cell lines have been used in many studies [9]–[11]. However, transformed lymphoblasts may undergo changes in their mRNA levels compared with the original peripheral blood lymphocyte sample because of biological noise and *in vitro* artifacts (the levels of Epstein–Barr virus used to transform the cells, ATP levels, etc.), or even extreme clonal effects may compromise an ASE analysis. For these reasons, the use of bulk nontransformed cells or *ex vivo* cells is recommended for ASE assays [22]. All previously reported *TGFBR1* ASE studies used SNPs as genetic markers [8]–[14]. SNaPshot [8], [9], [11] and pyrosequencing [10], [12]–[14] technologies have been used to analyze SNPs, and have generated discrepancies in published *TGFBR1* ASE results. We selected an insertion/deletion polymorphism (rs11466445) associated with the ASE phenomenon [8] and studied it with capillary electrophoresis fragment analysis, which is the most appropriate methodology for the analysis of this type of polymorphism.

We are aware of the limitations of this study. The sample size used in this study allowed us to detect susceptibility factors with a minor allele frequency of 8% with RR≥2 or ≤0.5, and with 80% power for the crude associations of simple factors. Therefore, the significant associations we found for the SNPs and haplotypes were underpowered. Furthermore, the size of this study sample did not allow us to detect the effects of rare alleles. However, our intention was to analyze the effects of the combination of common genetic factors. The sensitivity to detect genetic susceptibility is lower when the population is stratified. Therefore, the risk detected in the combined analysis of ASE and the H2 haplotype based on the available sample size is clearly underpowered. The specificity and sensitivity values for that association were 95% and 25%, respectively.

The expected frequencies for H2/ASE, when they were considered as independent factors, were significantly different from the observed frequencies in the patients (*P* = 0.013) but not in the controls (*P*>0.05; Table S8), suggesting that this combination of factors may have a deleterious effect.

The presence of a haplotype associated with ASE suggests a *cis* regulatory mechanism underlying ASE. Polymorphisms in the gene promoter, enhancer, transcription-factor-binding sites, splicing sites, RNA stability elements, or antisense RNAs might be involved in the underlying mechanistic dysfunction [24].

Epistatic interactions are difficult to detect unless their marginal effects are significant. There is also a statistical penalty conferred by large-scale multiple testing, which may make the identification of such interactions highly problematic. Therefore, further investigations using larger samples are required to ensure conclusive results.

The polymorphisms selected for studies of multifactorial genetic associations with diseases, such as the present one, are of critical importance. The high level of LD shown by the set of polymorphisms used in this work allowed us to dissect the polymorphisms that defined those subhaplotypes that are more strongly associated with the disease. A submaximal LD effect may hide potentially useful information that would be missed in those genetic epidemiology studies in which predefined tagSNPs are used. At this point, it is important to note that patterns of LD can differ markedly between populations [25].

All common SNPs identified to date with GWA scans confer modest cancer risks and the majority of susceptibility alleles have ORs of <1.5. Furthermore, in the reported GWA studies, only 12% of SNPs with MAFs of 5%–10% were tagged, indicating that these strategies are not optimally configured to identify low-frequency variants in this range of MAFs, some of which may have stronger effects [2]. Fifty percent of the *TGFBR1* intralocus polymorphisms (7/14) used in the present study had MAFs ranging from 8% to 10%, allowing the detection of new risk factors.

Haplotype-based approaches may have greater power than single-locus analyses when the SNPs are in strong LD with the risk *locus*. New data-mining approaches are being used to overcome potential complexities that arise in genetic studies from large numbers of haplotypes, offering more insight into the genetic risk factors associated with complex diseases [26].

Despite the limitations described above, our results suggest the importance of *TGFBR1* in the genetic susceptibility to so-called “sporadic” CRC. These results also offer a proof of concept for the existence of intralocus epistatic interactions between common variants associated with CRC susceptibility. Therefore, a detailed map of genetic interactions is required for more accurate risk assessment, which should allow cancer prevention strategies to be targeted, and increasingly influence cancer treatments.

## Supporting Information

Figure S1 **Location of the analyzed polymorphisms at the** ***TGFBR1 locus*** **.** Thirteen SNPs and an insertion deletion polymorphism (rs11466445) at the *TGFBR1 locus* were genotyped. Six polymorphisms were intragenic, five were located upstream from the gene, and three were downstream from the gene.
(TIF)

Table S1 **Allelic frequencies of the** ***TGFBR1*** **polymorphisms by group.** (CRC: colorectal cancer; C: controls).
(DOC)

Table S2 **Genotypic frequencies of the** ***TGFBR1*** **polymorphisms by group.** (CRC: colorectal cancer; C: controls).
(DOC)

Table S3 **Linkage disequilibrium between the analyzed polymorphisms at the** ***TGFBR1*** **locus (D′ value).**
(DOC)

Table S4 **Definition of the** ***TGFBR1*** **haplotypes found in the present study.**
(DOC)

Table S5 **Frequencies of the** ***TGFBR1*** **haplotypes in the patients and controls groups.** (CRC: colorectal cancer; C: controls).
(DOC)

Table S6 **Receiver operating curve analysis to determine the cutoff values for** ***TGFBR1*** **ASE.** (CRC: colorectal cancer; C: controls; ASE: allele-specific expression; YI: Youden's Index).
(DOC)

Table S7 **Association between** ***TGFBR1*** **ASE and CRC: crude and stratified analyses.** (CRC: colorectal cancer; C: controls; ASE: allele-specific expression; OR: odds ratio; CI: confidence interval).
(DOC)

Table S8 **Observed and expected frequencies of the** ***TGFBR1*** **H2 haplotype and** ***TGFBR1*** **ASE in patients and controls.**
(DOC)
