# Supplementary figures and images for: TGFBR1 Intralocus Epistatic Interaction as a Risk Factor for Colorectal Cancer
Source: PLoS One. 2012 Jan 23;7(1):e30812. doi: 10.1371/journal.pone.0030812 (PMC3264637; doi:10.1371/journal.pone.0030812)

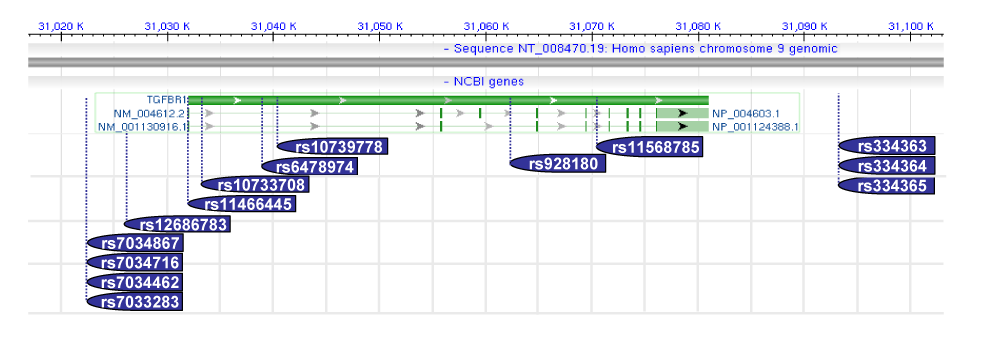

Supplement: Figure S1 — Location of the analyzed polymorphisms at the TGFBR1 locus . Thirteen SNPs and an insertion deletion polymorphism (rs11466445) at the TGFBR1 locus were genotyped. Six polymorphisms were intragenic, five were located upstream from the gene, and three were downstream from the gene. (TIF) [file pone.0030812.s001.tif]
