# Supplementary material for: TGFBR1 Intralocus Epistatic Interaction as a Risk Factor for Colorectal Cancer
Source: PLoS One. 2012 Jan 23;7(1):e30812. doi: 10.1371/journal.pone.0030812 (PMC3264637; doi:10.1371/journal.pone.0030812)
Supplement: Table S1 — Allelic frequencies of the TGFBR1 polymorphisms by group. (CRC: colorectal cancer; C: controls). (DOC) [file pone.0030812.s002.doc]

| **Polymorphism** | **CRC** | **C** |
| --- | --- | --- |
| **rs7033283** | (n=804) | (n=726) |
| G | 0.9254 | 0.9063 |
| A | 0.0746 | 0.0937 |
| **rs7034462** | (n=804) | (n=738) |
| C | 0.9254 | 0.9079 |
| T | 0.0746 | 0.0921 |
| **rs7034716** | (n=806) | (n=726) |
| C | 0.7146 | 0.6970 |
| T | 0.2854 | 0.3030 |
| **rs7034867** | (n=804) | (n=750) |
| C | 0.9181 | 0.9107 |
| A | 0.0819 | 0.0893 |
| **rs12686783** | (n=808) | (n=742) |
| C | 0.9233 | 0.9111 |
| T | 0.0767 | 0.0889 |
| **rs11466445** | (n=810) | (n=754) |
| *9A | 0.9173 | 0.9072 |
| *6A | 0.0827 | 0.0928 |
| **rs10733708** | (n=778) | (n=732) |
| G | 0.7018 | 0.6831 |
| A | 0.2982 | 0.3169 |
| **rs6478974** | (n=772) | (n=730) |
| T | 0.5389 | 0.5301 |
| A | 0.4611 | 0.4699 |
| **rs10739778** | (n=810) | (n=742) |
| A | 0.6494 | 0.6199 |
| C | 0.3506 | 0.3801 |
| **rs928180** | (n=790) | (n=570) |
| A | 0.9367 | 0.9088 |
| G | 0.0633 | 0.0912 |
| **rs11568785** | (n=810) | (n=752) |
| A | 0.9296 | 0.9189 |
| G | 0.0704 | 0.0811 |
| **rs334363** | (n=808) | (n=752) |
| A | 0.6572 | 0.6343 |
| C | 0.3428 | 0.3657 |
| **rs334364** | (n=806) | (n=734) |
| C | 0.6600 | 0.6362 |
| T | 0.3400 | 0.3638 |
| **rs334365** | (n=792) | (n=718) |
| G | 0.6553 | 0.6240 |
| A | 0.3447 | 0.3760 |
