# Supplementary material for: TGFBR1 Intralocus Epistatic Interaction as a Risk Factor for Colorectal Cancer
Source: PLoS One. 2012 Jan 23;7(1):e30812. doi: 10.1371/journal.pone.0030812 (PMC3264637; doi:10.1371/journal.pone.0030812)
Supplement: Table S2 — Genotypic frequencies of the TGFBR1 polymorphisms by group. (CRC: colorectal cancer; C: controls). (DOC) [file pone.0030812.s003.doc]

| **Polymorphism** | **CRC** | | **C** | |
| --- | --- | --- | --- | --- |
| **rs7033283** | (n=402) | | (n=363) | |
| GG | 345 | 85.82% | 297 | 81.82% |
| AG | 54 | 13.43% | 64 | 17.63% |
| AA | 3 | 0.75% | 2 | 0.55% |
| **rs7034462** | (n=402) | | (n=369) | |
| CC | 345 | 85.82% | 303 | 82.11% |
| CT | 54 | 13.43% | 64 | 17.34% |
| TT | 3 | 0.75% | 2 | 0.54% |
| **rs7034716** | (n=403) | | (n=363) | |
| CC | 214 | 53.10% | 171 | 47.11% |
| CT | 148 | 36.72% | 164 | 45.18% |
| TT | 41 | 10.17% | 28 | 7.71% |
| **rs7034867** | (n=403) | | (n=375) | |
| CC | 340 | 84.37% | 310 | 82.67% |
| AC | 60 | 14.89% | 63 | 16.80% |
| AA | 3 | 0.74% | 2 | 0.53% |
| **rs12686783** | (n=404) | | (n=371) | |
| CC | 345 | 85.40% | 308 | 83.02% |
| CT | 56 | 13.86% | 60 | 16.17% |
| TT | 3 | 0.74% | 3 | 0.81% |
| **rs11466445** | (n=521) | | (n=404) | |
| *9A/*9A | 442 | 84.84% | 334 | 82.67% |
| *9A/*6A | 72 | 13.82% | 67 | 16.58% |
| *6A/*6A | 7 | 1.34% | 3 | 0.74% |
| **rs10733708** | (n=389) | | (n=366) | |
| GG | 199 | 51.16% | 168 | 45.90% |
| AG | 148 | 38.05% | 164 | 44.81% |
| AA | 42 | 10.80% | 34 | 9.29% |
| **rs6478974** | (n=386) | | (n=365) | |
| TT | 116 | 30.05% | 101 | 27.67% |
| AT | 184 | 47.67% | 185 | 50.68% |
| AA | 86 | 22.28% | 79 | 21.64% |
| **rs10739778** | (n=405) | | (n=371) | |
| AA | 178 | 43.95% | 138 | 37.20% |
| AC | 170 | 41.98% | 184 | 49.60% |
| CC | 57 | 14.07% | 49 | 13.21% |
| **rs928180** | (n=395) | | (n=285) | |
| AA | 348 | 88.10% | 236 | 82.81% |
| AG | 44 | 11.14% | 46 | 16.14% |
| GG | 3 | 0.76% | 3 | 1.05% |
| **rs11568785** | (n=405) | | (n=376) | |
| AA | 350 | 86.42% | 317 | 84.31% |
| AG | 53 | 13.09% | 57 | 15.16% |
| GG | 2 | 0.49% | 2 | 0.53% |
| **rs334363** | (n=404) | | (n=376) | |
| AA | 180 | 44.55% | 147 | 39.10% |
| AC | 171 | 42.33% | 183 | 48.67% |
| CC | 53 | 13.12% | 46 | 12.23% |
