# Supplementary material for: TGFBR1 Intralocus Epistatic Interaction as a Risk Factor for Colorectal Cancer
Source: PLoS One. 2012 Jan 23;7(1):e30812. doi: 10.1371/journal.pone.0030812 (PMC3264637; doi:10.1371/journal.pone.0030812)
Supplement: Table S3 — Linkage disequilibrium between the analyzed polymorphisms at the TGFBR1 locus (D′ value). (DOC) [file pone.0030812.s004.doc]

|  | rs7034462 | rs7034716 | rs7034867 | rs12686783 | rs11466445 | rs10733708 | rs6478974 | rs10739778 | rs928180 | rs11568785 | rs334363 | rs334364 | rs334365 |
| --- | --- | --- | --- | --- | --- | --- | --- | --- | --- | --- | --- | --- | --- |
| rs7033283 | 1.000 | 1.000 | 0.886 | 1.000 | 0.858 | 1.000 | 1.000 | 1.000 | 0.965 | 1.000 | 0.797 | 0.797 | 1.000 |
| rs7034462 |  | 1.000 | 0.886 | 1.000 | 0.858 | 1.000 | 1.000 | 1.000 | 0.965 | 1.000 | 0.797 | 0.797 | 1.000 |
| rs7034716 |  |  | 1.000 | 0.979 | 0.958 | 1.000 | 1.000 | 1.000 | 0.954 | 1.000 | 0.918 | 0.918 | 0.979 |
| rs7034867 |  |  |  | 0.886 | 1.000 | 1.000 | 1.000 | 1.000 | 0.965 | 1.000 | 1.000 | 1.000 | 1.000 |
| rs12686783 |  |  |  |  | 0.858 | 1.000 | 1.000 | 1.000 | 0.965 | 1.000 | 0.800 | 0.800 | 1.000 |
| rs11466445 |  |  |  |  |  | 0.958 | 0.980 | 0.954 | 0.965 | 1.000 | 0.955 | 0.955 | 0.954 |
| rs10733708 |  |  |  |  |  |  | 0.991 | 0.913 | 0.953 | 1.000 | 0.894 | 0.894 | 0.953 |
| rs6478974 |  |  |  |  |  |  |  | 0.617 | 1.000 | 1.000 | 0.610 | 0.616 | 0.628 |
| rs10739778 |  |  |  |  |  |  |  |  | 0.949 | 1.000 | 0.988 | 0.988 | 0.989 |
| rs928180 |  |  |  |  |  |  |  |  |  | 1.000 | 1.000 | 1.000 | 1.000 |
| rs11568785 |  |  |  |  |  |  |  |  |  |  | 1.000 | 1.000 | 1.000 |
| rs334363 |  |  |  |  |  |  |  |  |  |  |  | 1.000 | 0.994 |
| rs334364 |  |  |  |  |  |  |  |  |  |  |  |  | 1.000 |
