# Supplementary material for: TGFBR1 Intralocus Epistatic Interaction as a Risk Factor for Colorectal Cancer
Source: PLoS One. 2012 Jan 23;7(1):e30812. doi: 10.1371/journal.pone.0030812 (PMC3264637; doi:10.1371/journal.pone.0030812)
Supplement: Table S4 — Definition of the TGFBR1 haplotypes found in the present study. (DOC) [file pone.0030812.s005.doc]

| **Haplotype** | |  | **Haplotype** | |
| --- | --- | --- | --- | --- |
| H1 | ATTAT***6**ATCGGCTA |  | H15 | GCCCT***9**ATCAACTA |
| H2 | GCCCC***9**GTAAAACG |  | H16 | GCCCC***9**GTAGACTA |
| H3 | GCCCC***9**AAAAACG |  | H17 | GCCCC***9**GTCAAACG |
| H4 | GCTCC***9**ATCAACTA |  | H18 | GCCCC***9**AAAAAACG |
| H5 | GCCCC***9**GACAACTA |  | H19 | GCCCC***6**GAAAAACG |
| H6 | GCTAC***6**ATCAACTA |  | H20 | GCCCC***6**GTCGGCTA |
| H7 | GCCCC***9**GACAACCG |  | H21 | ATTATT***6**TCGGCTA |
| H8 | GCTCC***9**ATCAAACG |  | H22 | GCTCC***6**ATCAACTA |
| H9 | GCCCC***9**GAAAACTA |  | H23 | GCTAC***9**ATCAACTA |
| H10 | GCTCC***9**ATAAACTA |  | H24 | ATTAT***6**ATCGACTA |
| H11 | GCCCC***9**ATCAACTA |  | H25 | GCTCC***9**GAAAAACG |
| H12 | GCCCC***9**GTCAACTA |  | H26 | GCCCC***9**ATCAACCG |
| H13 | ATTCT***9**ATCAAACA |  | H27 | GCTCC***9**AACAACTA |
| H14 | GCCCC***9**ATAAAACG |  |  |  |
