# Supplementary material for: TGFBR1 Intralocus Epistatic Interaction as a Risk Factor for Colorectal Cancer
Source: PLoS One. 2012 Jan 23;7(1):e30812. doi: 10.1371/journal.pone.0030812 (PMC3264637; doi:10.1371/journal.pone.0030812)
Supplement: Table S5 — Frequencies of the TGFBR1 haplotypes in the patients and controls groups. (CRC: colorectal cancer; C: controls). (DOC) [file pone.0030812.s006.doc]

| **Haplotype** | **CRC** | | **C** | |
| --- | --- | --- | --- | --- |
| **H1** | 54 | 6.67% | 61 | 8.10% |
| **H2** | 195 | 24.07% | 156 | 20.72% |
| **H3** | 328 | 40.49% | 303 | 40.24% |
| **H4** | 153 | 18.89% | 151 | 20.05% |
| **H5** | 46 | 5.68% | 50 | 6.64% |
| **H6** | 9 | 1.11% | 7 | 0.93% |
| **H7** | 1 | 0.12% | 1 | 0.13% |
| **H8** | 3 | 0.37% | 3 | 0.40% |
| **H9** | 1 | 0.12% | 0 | 0.00% |
| **H10** | 1 | 0.12% | 0 | 0.00% |
| **H11** | 3 | 0.37% | 2 | 0.27% |
| **H12** | 0 | 0.00% | 1 | 0.13% |
| **H13** | 4 | 0.49% | 9 | 1.20% |
| **H14** | 0 | 0.00% | 3 | 0.40% |
| **H15** | 1 | 0.12% | 1 | 0.13% |
| **H16** | 0 | 0.00% | 2 | 0.27% |
| **H17** | 1 | 0.12% | 1 | 0.13% |
| **H18** | 0 | 0.00% | 1 | 0.13% |
| **H19** | 0 | 0.00% | 2 | 0.27% |
| **H20** | 1 | 0.12% | 0 | 0.00% |
| **H21** | 2 | 0.25% | 0 | 0.00% |
| **H22** | 1 | 0.12% | 0 | 0.00% |
| **H23** | 1 | 0.12% | 0 | 0.00% |
| **H24** | 2 | 0.25% | 0 | 0.00% |
| **H25** | 1 | 0.12% | 0 | 0.00% |
| **H26** | 1 | 0.12 | 0 | 0.00% |
| **H27** | 1 | 0.12 | 0 | 0.00% |
