# Supplementary material for: TGFBR1 Intralocus Epistatic Interaction as a Risk Factor for Colorectal Cancer
Source: PLoS One. 2012 Jan 23;7(1):e30812. doi: 10.1371/journal.pone.0030812 (PMC3264637; doi:10.1371/journal.pone.0030812)
Supplement: Table S6 — Receiver operating curve analysis to determine the cutoff values for TGFBR1 ASE. (CRC: colorectal cancer; C: controls; ASE: allele-specific expression; YI: Youden's Index). (DOC) [file pone.0030812.s007.doc]

| **Allele** | **ASE** |  |  |  |  |  |  |
| --- | --- | --- | --- | --- | --- | --- | --- |
| **proportion** | **cutoff** | **ASE** | **CRC** | **C** | **Sensitivity** | **Specificity** | **YI** |
| **(*9A/*6A)** | **ratios** |  |  |  |  |  |  |
| 50:50 | 1 | Negatives | 0 | 0 | 1,000 | 0,000 | 0,000 |
|  |  | Positives | 61 | 67 |  |  |  |
|  |  |  |  |  |  |  |  |
| 49:51-51:49 | <0,96 or >1,04 | Negatives | 13 | 9 | 0,787 | 0,134 | -0,08 |
|  |  | Positives | 48 | 58 |  |  |  |
|  |  |  |  |  |  |  |  |
| 48:52-52:48 | <0,92 or >1,08 | Negatives | 20 | 23 | 0,672 | 0,343 | 0,015 |
|  |  | Positives | 41 | 44 |  |  |  |
|  |  |  |  |  |  |  |  |
| 47:53-53:47 | <0,89 or >1,17 | Negatives | 35 | 40 | 0,426 | 0,597 | -0,03 |
|  |  | Positives | 26 | 27 |  |  |  |
|  |  |  |  |  |  |  |  |
| 46:54-54:46 | <0,85 or >1,13 | Negatives | 35 | 40 | 0,426 | 0,597 | 0,023 |
|  |  | Positives | 26 | 27 |  |  |  |
|  |  |  |  |  |  |  |  |
| 45:55- 55:45 | <0,82 or >1,22 | Negatives | 39 | 49 | 0,361 | 0,731 | 0,092 |
|  |  | Positives | 22 | 18 |  |  |  |
|  |  |  |  |  |  |  |  |
| 44:56- 56:44 | <0,78 or >1,27 | Negatives | 43 | 56 | **0,295** | **0,836** | **0,131** |
|  |  | Positives | 18 | 11 |  |  |  |
|  |  |  |  |  |  |  |  |
| 43:57- 57:43 | <0,75 or >1,32 | Negatives | 47 | 57 | 0,230 | 0,851 | 0,08 |
|  |  | Positives | 14 | 10 |  |  |  |
|  |  |  |  |  |  |  |  |
| 42:58- 58:42 | <0,72 or >1,38 | Negatives | 51 | 60 | 0,164 | 0,896 | 0,059 |
|  |  | Positives | 10 | 7 |  |  |  |
|  |  |  |  |  |  |  |  |
| 41:59- 59:41 | <0,69 or >1,44 | Negatives | 52 | 63 | 0,148 | 0,940 | 0,088 |
|  |  | Positives | 9 | 4 |  |  |  |
|  |  |  |  |  |  |  |  |
| 40:60- 60:40 | <0,67 or >1,50 | Negatives | 52 | 64 | 0,148 | 0,955 | 0,103 |
|  |  | Positives | 9 | 3 |  |  |  |
|  |  |  |  |  |  |  |  |
| 39:61- 61:39 | <0,64 or >1,56 | Negatives | 57 | 66 | 0,066 | 0,985 | 0,051 |
|  |  | Positives | 4 | 1 |  |  |  |
|  |  |  |  |  |  |  |  |
| 36:64- 64:36 | <0,56 or >1,77 | Negatives | 58 | 67 | 0,049 | 1,000 | 0,049 |
|  |  | Positives | 3 | 0 |  |  |  |
|  |  |  |  |  |  |  |  |
| 30:70- 70:30 | <0,43 or >2,33 | Negatives | 59 | 67 | 0,033 | 1,000 | 0,033 |
|  |  | Positives | 2 | 0 |  |  |  |
|  |  |  |  |  |  |  |  |
| 25:75- 75:25 | <0,33 or >3,00 | Negatives | 60 | 67 | 0,016 | 1,000 | 0,016 |
|  |  | Positives | 1 | 0 |  |  |  |
|  |  |  |  |  |  |  |  |
| 10:90-90:10 | <0,11 or >9,00 | Negatives | 61 | 67 | 0,000 | 1,000 | 0,000 |
|  |  | Positives | 0 | 0 |  |  |  |
