# Supplementary material for: TGFBR1 Intralocus Epistatic Interaction as a Risk Factor for Colorectal Cancer
Source: PLoS One. 2012 Jan 23;7(1):e30812. doi: 10.1371/journal.pone.0030812 (PMC3264637; doi:10.1371/journal.pone.0030812)
Supplement: Table S7 — Association between TGFBR1 ASE and CRC: crude and stratified analyses. (CRC: colorectal cancer; C: controls; ASE: allele-specific expression; OR: odds ratio; CI: confidence interval). (DOC) [file pone.0030812.s008.doc]

|  | **CRC** | **C** | **OR** | **95% CI** | ***P*** |
| --- | --- | --- | --- | --- | --- |
|  |  |  |  |  |  |
| ASE positives | 18 | 11 |  |  |  |
| ASE negatives | 54 | 56 | 1.697 | (0.74-3.87) | 0.213 |
|  |  |  |  |  |  |
| **Sex** |  |  |  |  |  |
| Men | n=36 | n=21 |  |  |  |
| ASE positive | 10 | 1 |  |  |  |
| ASE negative | 26 | 20 | 7.692 | (1.14-49.56) | 0.076 |
|  |  |  |  |  |  |
| Women | n=23 | n=43 |  |  |  |
| ASE positive | 6 | 10 |  |  |  |
| ASE negative | 17 | 33 | 1.165 | (0.37-3.65) | 0.798 |
|  |  |  |  |  |  |
| **Age** |  |  |  |  |  |
| >67 years | n=33 | n=33 |  |  |  |
| ASE positive | 10 | 3 |  |  |  |
| ASE negative | 23 | 30 | 4.348 | (1.14-16.29) | 0.064 |
|  |  |  |  |  |  |
| ≤67 years | n=26 | n=31 |  |  |  |
| ASE positive | 6 | 8 |  |  |  |
| ASE negative | 20 | 23 | 0.863 | (0.26-2.82) | 0.812 |
|  |  |  |  |  |  |
| **Location** | Proximal | Distal |  |  |  |
|  | n=12 | n=37 |  |  |  |
| ASE positive | 6 | 9 |  |  |  |
| ASE negative | 6 | 28 | 3.111 | (0.84-11.69) | 0.094 |
|  |  |  |  |  |  |
|  |  |  |  |  |  |
| **Stage** | I and II | III and IV |  |  |  |
|  | n=23 | n=13 |  |  |  |
| ASE positive | 8 | 0 |  |  |  |
| ASE negative | 15 | 13 | inf | (1.62-inf) | 0.016 |
