# Supplementary material for: TGFBR1 Intralocus Epistatic Interaction as a Risk Factor for Colorectal Cancer
Source: PLoS One. 2012 Jan 23;7(1):e30812. doi: 10.1371/journal.pone.0030812 (PMC3264637; doi:10.1371/journal.pone.0030812)
Supplement: Table S8 — Observed and expected frequencies of the TGFBR1 H2 haplotype and TGFBR1 ASE in patients and controls. (DOC) [file pone.0030812.s009.doc]

|  |  | **Expected** | **Observed** |  |
| --- | --- | --- | --- | --- |
|  | **Frequency** | **frequency** | **frequency** | ***P*** |
| **CRC** | | | | |
| H2 | 0,241 |  |  |  |
| ASE | 0,250 | 0,060 | 0,333 | 0,013 |
| **C** | | | | |
| H2 | 0,207 |  |  |  |
| ASE | 0,164 | 0,034 | 0,159 | 0,056 |
